# *ColorI-DT*: An open-source tool for the quantitative evaluation of differences in microscopy color images

**DOI:** 10.1016/j.csbj.2025.06.019

**Published:** 2025-06-09

**Authors:** Filippo Piccinini, Michele Tritto, Jae-Chul Pyun, Misu Lee, Bongseop Kwak, Bosung Ku, Nicola Normanno, Gastone Castellani

**Affiliations:** a IRCCS Istituto Romagnolo per lo Studio dei Tumori (IRST) “Dino Amadori”, Via Piero Maroncelli 40, Meldola, FC 47014, Italy; b Department of Medical and Surgical Sciences (DIMEC), University of Bologna, via G. Massarenti 9, Bologna 40138, Italy; c Next2U Srl, Via dei Peligni 137, Pescara 65127, Italy; d Department of Materials Science and Engineering, Yonsei University, 50 Yonsei-ro, Seodaemun-gu, Seoul, Republic of Korea; e Division of Life Sciences, College of Life Science and Bioengineering, Incheon National University, 119 Academy-ro, Songdo-dong, Yeonsu-gu, Incheon, Republic of Korea; f Institute for New Drug Development, College of Life Science and Bioengineering, Incheon National University, 119 Academy-ro, Songdo-dong, Yeonsu-gu, Incheon, Republic of Korea; g College of Medicine, Dongguk University, 27 Dongguk-ro, Ilsandong-gu,, Goyang-si, Gyeonggi-do, Republic of Korea; h Central R&D Center, Medical and Bio Decision (MBD) Co., Ltd., B-8F, 9F 145, Gwanggyo-ro, Yeongtong-gu, Suwon-si, Gyeonggi-do, Suwon, Republic of Korea; i IRCCS Azienda Ospedaliero-Universitaria di Bologna S.Orsola, Via Giuseppe Massarenti 9, Bologna 40138, Italy

**Keywords:** Oncology, Microscopy, Image Processing, Color Spaces, Quantitative Metrics

## Abstract

In several fields, quantitatively comparing color images is crucial. For instance, this is important in Histopathology, where different microscopes/cameras are typically used for visualizing patient samples by causing significant color variation. No ground-truth metric exists for estimating differences between pairs of color images. A range of possible solutions is available but there is no existing open-source tool that allow clinicians and researchers to apply these metrics to microscopy images through an intuitive, easy-to-use software. In this work, we developed *Color Image Difference Tool* (*ColorI-DT*), an open-source tool for measuring quantitative differences between color images of the same subject acquired under different settings. Thanks to a user-friendly graphical user interface, it allows the selection of a pair of color images and a metric from a list of available options, and produces an output 2D pixel-wise color difference matrix between corresponding pixels in the input images. The metrics currently implemented are: (*1*) Euclidean ΔE; (*2*) International Commission on Illumination (CIE) 76 (Luv); (*3*) CIE76 (Lab); (*4*) CIE94; (*5*) CIE00; (*6*) Colour Measurement Committee (CMC). To demonstrate how to use the tool, microscopy images with a predominant color in the red, green, or blue channel were used. In particular, we checked which among the 6 metrics displays the most predictable and linear behavior in the case of controlled primary color alterations. For more pronounced color adjustments, a qualitative comparison would be likely sufficient for analyzing color differences, as a quantitative tool may become unreliable due to the inherent limitations of the implemented metrics.

## Highlights

• Comparing color images is crucial in fields like Oncology and Histopathology.
• ColorI-DT, an open-source tool to measure color differences between image pairs.
• The metrics implemented are Euclidean ΔE, CIE76 (Luv), CIE76 (Lab), CIE94, CIE00, CMC.
• ColorI-DT generates 2D pixel-wise color difference matrices.
• The tool was validated using images with predominant red, green, or blue channels.
• ColorI-DT is freely available for MAC, Linux, and Windows

## Graphical Abstract

**Figure:**
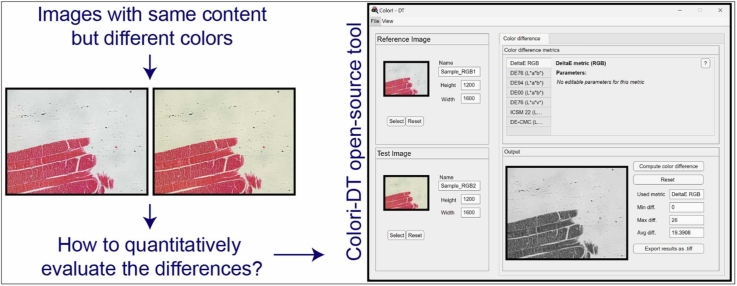

## 1 Introduction

In different domains, the possibility to quantitatively compare color images depicting the same subject but with varying levels of noise or color palettes is a fundamental requirement [1]. In fields like Oncology, particularly within Histopathology, color images play a crucial role because they are used to highlight various cellular and subcellular structures in biological samples stained with specific dyes. For example, hematoxylin and eosin (H&E) staining is one of the most common techniques for visualizing cells in brightfield. Precisely, hematoxylin stains cell nuclei in blue, and eosin stains cytoplasm and extracellular matrix in pink. This color contrast helps pathologists to examine tissues, identifying cancer cells, inflammation, or/and other pathological conditions [newA].

**Fig. 1:**
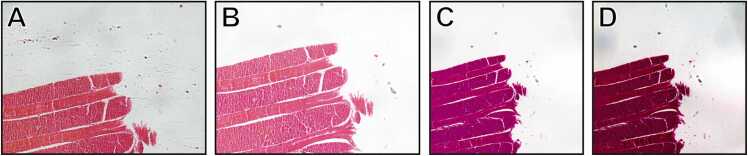
Histological sample images with a predominant red color of a commercial human tongue biopsy (Salmoiraghi & Viganò, Legnago, Veneto, Italy) acquired using different microscopes and settings. (**A**) Image acquired using an Olympus IX51 microscope, equipped with a Nikon DS-Vi1 RGB camera and a 10x brightfield objective, without applying any image post-processing; (**B**) Image acquired using the same equipment of **A**, but applying a white balancing correction through the acquisition software; (**C**) Image acquired using a Nikon A1R microscope, equipped with a Nikon DS-Fi3 RGB camera and a 10x brightfield objective, without applying any image post-processing; (**D**) Image acquired using the same equipment of **C**, but applying an intense gamma correction through the acquisition software. Although the subject is easily recognizable, the resulting color palettes vary noticeably among the different images.

Typically, a variety of microscopes, cameras, or identical equipment with different configurations are routinely used to examine patient samples, leading to significant color variation across different images (Fig. 1) [2] and across different datasets [newB]. The possibility to quantitatively compare color images is critical in scenarios involving the calibration of diagnostic tools or the comparison of similar imaging technologies, for instance different microscope settings [3]. By quantitatively measuring color discrepancies between reference and target images, it is possible guiding microscope setup optimization fine-tuning parameters such as illumination, exposure, white balance, and camera settings to minimize variation and achieve more consistent image acquisition. This is especially beneficial for microscopists working with multiple imaging systems or preparing for multi-center studies, where maintaining color consistency is crucial [newC].

Several solutions have been proposed in the literature for quantifying color disparities between pairs of images depicting the same object but acquired with different settings. However, despite the crucial nature of precise comparisons, a universally accepted metric is currently lacking. For comparing monochromatic images, the most common quantitative metrics can be grouped in the following categories [4]: (*a*) Generic signal analysis metrics, for instance the Mean Square Error (MSE) or its variations like the Root Mean Square Error (RMSE) [5]; (*b*) Image signal analysis metrics, like the Signal-to-Noise Ratio (SNR) or the Peak Signal-to-Noise Ratio (PSNR) [6]; (*c*) Image visual analysis metrics, for instance, the Universal Quality Index (UQI) [7]; (*d*) Generic similarity of data metrics, like the Jaccard Similarity Coefficient, also simply called Jaccard Index or Intersection over Union (IoU) [8]. These metrics typically evaluate factors such as contrast, sharpness, and noise levels to determine the overall quality of an image [9]. They have different pros and cons and it is important to consider the specific image application when choosing the comparison strategy. However, while extensive research has focused on quantitatively comparing metrics for monochromatic images, studies comparing different strategies for quantitatively computing differences in color images, for instance in RGB images (the most common color image format with primary Red, Green, and Blue channels), are still limited [10]. Precisely, despite the normalization of color images [newD] have been shown to be fundamental for various applications [newE, newF], a tool making the state-of-the-art color metrics easily accessible is still missing.

In this work, we presented the *Color Image Difference Tool* (*ColorI-DT*), an open-source tool designed to easily measure quantitative differences between pairs of color images of the same subject but acquired in different conditions by selecting one of the metrics currently published for comparing color images. It is important to note that the implemented metrics have already been published and validated: no new metric is proposed in this regard. The tool is written in *MATLAB* (The MathWorks, Inc., MA, USA). It provides a user-friendly graphical user interface (GUI) that allows the user to select the images to be compared and the metric to be used, and generates as output a 2D error matrix. Specifically, *ColorI-DT* operates on pairs of input 8-bit RGB “.jpg”, “.jpeg”, “.png”, and “.tiff” images with identical dimensions. Obviously, images of different dimensions or acquired with different cameras can be compared using *ColorI-DT*, but in this case, they must be pre-aligned and scaled to the same size using, for instance, the open-source tool *DS4H Image Alignment* [11]. The implemented metrics work on different color spaces [12], mainly: (*I*) the standard RGB [13]; (*II*) Luv ("L" represents lightness, the perceived brightness of the color, "u" the chromaticity, the position of the color, along the red-green axis, and "v" the chromaticity along the yellow-blue axis), often referred to as CIELUV, where CIE stands for International Commission on Illumination (in French, “Commission Internationale de l’Éclairage”) [14]; (*III*) Lab ("L" represents lightness, "a" the position of the color on the green-red axis, and "b" the position of the color on the blue-yellow axis), also written as CIELAB [15]; (*IV*) LCh ("L" represents lightness, "C" chroma, the colorfulness or saturation of a color, and "h" meaning hue, the dominant wavelength of the color, or in simpler terms, the perceived color family), that is a cylindrical representation of the Lab color space [16]. However, the users do not interact with the color space internally utilized by the metric. They simply receive as output a 2D 8-bit “.tiff” image created by calculating the pixel-wise color difference between corresponding pixels in the input images. The metrics currently implemented in *ColorI-DT* are: (1) Euclidean ΔE (working in the standard RGB color space); (2) CIE76 (Luv); (3) CIE76 (Lab); (4) CIE94 (Lab); (5) CIE00 (Lab); (6) CMC (LCh).

To test the tool, we used several brightfield microscopy images. First, we evaluated which among the different metrics exhibits the most predictable and linear behavior in response to controlled color alterations. For more pronounced chromaticity adjustments, a qualitative comparison is likely sufficient for analyzing color differences, as a quantitative tool may become unreliable and, therefore, of limited practical use. The analysis started by selecting representative images with a primary color and creating a testset of synthetic images corrupted by small, controlled image perturbations, such as those caused by minor changes in lighting or staining, which are common in microscopy. Precisely, we reduced the intensity of the RGB channels (one at a time) by 2 %, 5 %, and 10 % for 3 reference images, each dominated by either red (R), green (G), or blue (B). This produced 3 sets of 9 synthetic images, which were then quantitatively compared to the originals using the 6 color difference metrics. A total of 162 2D error matrices was analyzed with histogram-based statistics (i.e. mean, variance, skewness, kurtosis, and entropy). Principal Component Analysis (PCA) was used to cluster the metrics and identify patterns. Correlation heatmaps were created to examine the relationship between the mean, variance, and controlled color intensity changes. Finally, linear regression was used to evaluate how color variations affected the statistical features of the output images. With regards to the linear regression, it is worth noting that the standard RGB space is not supposed to be linear with respect to any perceptual color space [17]. However, given the synthetic images used in the experiments with a limited percentage reduction of a single RGB channel at a time, it is reasonable to assume a linear relationship.

The results obtained revealed that several metrics available in the literature, exhibit strong correlations to small, controlled alterations of the values of the primary color channel of RGB images. Briefly, CIE76 (Luv) outperformed CIE76 (Lab), and the most-adaptable CIE00 and CIE94 offer the best perceptual accuracy, addressing non-uniformities in earlier metrics like CIE76s. CMC is particularly useful in fields where specific lightness and chroma tolerances are critical and Euclidean ΔE is the simplest and least computationally demanding. However, the parameter-dependent CIE00 emerged as the most reliable metric in the majority of cases, followed by CIE94 and the robust parameter-independent CIE76 (Luv).

In conclusion, *ColorI*-DT facilitates the comparison of different color images of the same subject offering a powerful tool for users interested to (*a*) visualize pairs of color images, (*b*) select a color difference metric along with appropriate input parameters, and (*c*) calculate pixel-wise differences visualized as a grayscale 2D matrix. This tool represents a significant advancement in the field of color image comparison, providing to researchers and clinicians an user-friendly tool for quantitative color difference estimation. The *ColorI-DT MATLAB* source code, standalone applications (free-to-use without requiring any licenses) for all the main different operating systems (i.e. *Windows*, *macOS*, *Linux*), user documentation, video tutorial, and sample datasets are freely provided at: https://sourceforge.net/p/colori-dt/.

The next sections are organized as follows: Section 2 presents the details of the quantitative metrics implemented, the *ColorI-DT* architecture, and the testbed for experiments. Section 3 analyzes the results obtained testing *ColorI-DT*. Finally, Section 4 reports the main findings of the work together with future intentions.

## 2 Materials and methods

In the Sections, the mathematical equations of the 6 implemented color comparison metrics, the details of the *ColorI-DT*’s architecture, and the characteristics of the performed experiments are described.

### 2.1 Quantitative metrics

A function is classified as a distance metric if the following 4 mathematical criteria are satisfied: (*a*) *non-negativity*: a distance metric d(a,b) between any two points a and b must always be greater than or equal to zero. Practically: d(a,b)*≥* 0 for all a and b in the Cartesian plane; (*b*) *identity of indiscernible*: a distance metric must satisfy the condition d(a,b)= 0 if and only if a=b. In other words, the only way for the distance between two points to be zero is if the points are identical; (*c*) *symmetry*: a distance metric must satisfy the property d(a,b)=d(b,a) for all points a and b; (*d*) *triangle inequality*: for any three points a,b, and c, a distance metric must satisfy the property d(a,c)≤ d(a,b)+d(b,c) [18]. All the 6 implemented metrics satisfy the above 4 mathematical criteria and can then be defined as distance metrics. In mathematical terms, these 6 metrics are defined with an Euclidean difference in a 3D reference and a correction factor according to Eq. 1:

(1) $$\mathrm{metric}=\sqrt{{\left(\Delta x\right)}^{2}+{\left(\Delta y\right)}^{2}+{\left(\Delta z\right)}^{2}+f}$$

*x*, *y* and *z* are the coordinates of the color space. *f* a correction factor that some of the metrics have to compensate for specific non-linearities of the color space of application. Δx, Δy, and Δz are defined as the differences *x*_*2*_-*x*_*1*_, *y*_*2*_-*y*_*1*_, and *z*_*2*_-*z*_*1*_ between image *1* and image *2*, respectively. Importantly, all the implemented metrics are conceived to measure small color differences. Hence, no significance is attributable to higher values corresponding to perceptually different colors. In general, these metrics have a lower limit at zero and no upper limit. When applied to digital imaging, an upper limit appears as a result of the specific bit depth of the color quantization of the color space. Moreover, there is no direct relation between the values of color difference calculated in different color spaces, for instance, there is no direct conversion formula that can transform a color difference value from the CIELAB domain to CIELUV and *vice versa* [15]. **Supplementary Note 1** reports the *MATLAB* source-code lines of the implementation proposed for the single metrics.

The 6 metrics currently implemented in *ColorI-DT* are described by reporting for each metric: (*1*) a brief description, (*2*) mathematical equations, (*3*) the color space of application, (*4*) the main citation.

### 2.2 Euclidean ΔE

ΔE is a straightforward application of the Euclidean distance to the RGB color space. This metric is not perceptually accurate. However, it is generally used as the reference to compare color differences in the RGB color space, in which microscopy images are typically represented. The classical mathematical representation of the ΔE metric is reported in Eq. 2.

(2) $$\Delta E=\sqrt{{\left(\Delta R\right)}^{2}+{\left(\Delta G\right)}^{2}+{\left(\Delta B\right)}^{2}}$$

*R*, *G* and *B* are the values in the Red, Green and Blue channel, respectively. ΔR, ΔG, and ΔB, are defined as *R*_*2*_-*R*_*1*_, *G*_*2*_-*G*_*1*_, and *B*_*2*_-*B*_*1*_.

### 2.3 CIE76 (Lab)

The CIE76 (Lab) color difference metric is the first metric proposed in 1976 by CIE for the CIELAB color space [19]. Basically, it is a simple Euclidean distance defined in the Lab color space and not in the classical RGB one. The mathematical representation is reported in Eq. 3.

(3) $$\mathrm{CIE}76(\mathrm{Lab})=\sqrt{{\left(\Delta L\right)}^{2}+{\left(\Delta a\right)}^{2}+{\left(\Delta b\right)}^{2}}$$

*L*, *a* and *b* are the values in the respective channels. ΔL, Δa, and Δb, are defined as *L*_*2*_-*L*_*1*_, *a*_*2*_-*a*_*1*_, and *b*_*2*_-*b*_*1*_. However, this metric has been demonstrated to be not perceptually accurate and consequently does not perform adequately in measuring color differences. Accordingly, it has been replaced by more recent versions of this metric introducing some correction terms in the mathematical equation.

### 2.4 CIE76 (Luv)

The CIE76 (Luv) color difference metric, proposed by CIE for the CIELUV color space in 1976, has an analogous definition to the CIE76 (Lab) [19]. The metric is an Euclidean metric that operates in the CIELUV, a color space mostly used for additive color mixtures. The mathematical representation is reported in Eq. 4.

(4) $$\mathrm{CIE}76(\mathrm{Luv})=\sqrt{{\left(\Delta L\right)}^{2}+{\left(\Delta u\right)}^{2}+{\left(\Delta v\right)}^{2}}$$

*L*, *u* and *v* are the values in the respective channels. ΔL, Δu, and Δv, are defined as *L*_*2*_-*L*_*1*_, *u*_*2*_-*u*_*1*_, and *v*_*2*_-*v*_*1*_.

### 2.5 CIE94 (Lab)

The CIE94 color difference metric is an improvement of the CIE76 (Lab) metric that introduces weighting coefficients to correct the non-uniformities of the CIELAB color space [20]. The mathematical representation of CIE94 (Lab) is defined by Eq. 5, **Eq. 6**, Eq. 7, **Eq. 8**, and Eq. 9.

(5) $$\mathrm{CIE}94(\mathrm{Lab})=\sqrt{{\left(\frac{\Delta L}{k_{L}S_{L}}\right)}^{2}+{\left(\frac{\Delta C_{\mathrm{ab}}}{k_{C}S_{C}}\right)}^{2}+{\left(\frac{\Delta H_{\mathrm{ab}}}{k_{H}S_{H}}\right)}^{2}}$$

(6) $$\Delta C_{\mathrm{ab}}=C_{1}-C_{2};\;C_{1}=\sqrt{a_{1}^{2}+b_{1}^{2}};\;C_{2}=\sqrt{a_{2}^{2}+b_{2}^{2}}$$

(7) $$\Delta H_{\mathrm{ab}}=\sqrt{\Delta a^{2}+\Delta b^{2}-\Delta C_{\mathrm{ab}}^{2}}$$

(8) $$S_{C}=1+k_{1}C_{1}$$

(9) $$S_{H}=1+k_{2}C_{2}$$

ΔC represents the difference in chroma, ΔH the difference in hue. *S*_*L*_ is typically 1. The parameters *k*_L_, *k*_1_, and *k*_2_, are weighting parameters whose value depends on the application: for inks, paints and plastics *k*_L_ = 1, *k*_1_ = 0.045, and *k*_2_ = 0.015, for textile *k*_L_ = 2, *k*_1_ = 0.048, and *k*_2_ = 0.014 [21]. *k*_C_ and *k*_H_ are typically set to unity under the hypothesis of a standard colorimetric observer [22]. Advantages given by the metric CIE94 over the original CIE76 metrics include: (*I*) a better perceptual uniformity; (*II*) a measurement that directly involves chroma and hue; (*III*) parameters that allow for a degree of adaptation to industry-specific conditions. While improving perceptual uniformity, the CIE94 metric is still not completely uniform.

### 2.6 CIE00 (Lab)

The CIE00 metric, mostly reported in the literature with the extended version of the acronym, *i.e*. CIEDE2000, was developed by testing adaptations of the CIE94 (Lab) formula on samples with small color differences [23]. Notably, the *S*_*L*_, *S*_*H*_, and *S*_*C*_ weighting functions differ in definition from CIE94, and a rotational term *R*_*T*_ is introduced to account for interactions between chroma and hue in the blue region of the color space, achieving a closer agreement between the perception of color difference and the numerical difference values [24] [25]. The mathematical representation of CIE00 (Lab) is defined by Eq. 10, **Eq. 11**, Eq. 12, **Eq. 13**, Eq. 14, **Eq. 15**, and Eq. 16.

(10) $$\mathrm{CIE}00(\mathrm{Lab})=\sqrt{{\left(\frac{\Delta L}{k_{L}S_{L}}\right)}^{2}+{\left(\frac{\Delta C\prime }{k_{C}S_{C}}\right)}^{2}+{\left(\frac{\Delta H\prime }{k_{H}S_{H}}\right)}^{2}+R_{T}\left(\frac{\Delta C}{k_{C}S_{C}}\right)\left(\frac{\Delta H\prime }{k_{H}S_{H}}\right)}$$

(11) $${a\prime }_{1}=a_{1}+\frac{a_{1}}{2}\left(1-\sqrt{\frac{{\underset{¯}{C}}^{7}}{{\underset{¯}{C}}^{7}+\;25^{7}}}\right);{a\prime }_{2}=a_{2}+\frac{a_{1}}{2}\left(1-\sqrt{\frac{{\underset{¯}{C}}^{7}}{{\underset{¯}{C}}^{7}+\;25^{7}}}\right)$$

(12) $${\Delta C\prime ={C\prime }_{1}-{C\prime }_{2};\;C\prime }_{1}=\sqrt{{a\prime }_{1}^{2}+{b\prime }_{1}^{2}};{\;C\prime }_{2}=\sqrt{{a\prime }_{2}^{2}+{b\prime }_{2}^{2}}$$

(13) $$\underline{C}=\frac{C_{1}+C_{2}}{2};\underline{C}\prime =\frac{{C\prime }_{1}+{C\prime }_{2}}{2}\;$$

(14) $${h\prime }_{1}=\mathrm{atan}2(b_{1}+{a\prime }_{1})\mathrm{mod}360;{h\prime }_{2}=\mathrm{atan}2(b_{2}+{a\prime }_{2})\mathrm{mod}360;\;$$

$$\Delta h\prime ={h\prime }_{2}-{h\prime }_{1}\;(\mathrm{if}\left|h_{1}-h_{2}\right|\leq 180{}^{\circ});$$

$$\Delta h\prime ={h\prime }_{2}-{h\prime }_{1}+360{}^{\circ}\;(\mathrm{if}\left|h_{1}-h_{2}\right|>180{}^{\circ},{h\prime }_{2}\leq {h\prime }_{1});$$

(15) $$\Delta h\prime ={h\prime }_{2}-{h\prime }_{1}-360{}^{\circ}\;(\mathrm{if}\left|h_{1}-h_{2}\right|>180{}^{\circ},{h\prime }_{2}>{h\prime }_{1})\;$$

(16) $$\Delta H\prime =2\sqrt{{C\prime }_{1}{C\prime }_{2}}\sin\left(\frac{\Delta h\prime }{2}\right)$$

### 2.7 CMC (LCh)

The CMC (LCh) was developed in 1984 by the *Colour Measurement Committee* of the *Society of Dyers and Colourists* (SDC) on the cylindrical LCh formulation of the CIELAB color space. It was designed to improve upon the existing CIE76 (LAB). It calculates differences of the lightness, chroma and hue dimensions, and to lightness and chroma applies weighting functions that are designed to account for a specific the observation: the significance of variations in lightness, chroma, and hue to the overall perceived color difference, changes depending on where the color is located within the CIELAB space. The CMC (LCh) color difference formula creates an ellipsoidal tolerance region around a reference color. This region represents the acceptable color difference, and its size and shape can vary depending on the *l*:*c* ratio (where *l* and *c* define the desired weight for lightness and chroma in the equation of this color difference metric) and the position in the color space. The CMC (LCh) color difference formula is typically used for tolerancing color samples in the textile industry [26]. Common values for the *l*:*c* ratio are 2:1 for the acceptability of the color difference, 1:1 for the perceptibility of the color difference. The mathematical representation of CIE00 (Lab) is defined by Eq. 17, **Eq. 18**, Eq. 19, **Eq. 20**, Eq. 21, and **Eq. 22**.

(17) $$\mathrm{CMC}(\mathrm{LCh})=\sqrt{{\left(\frac{\Delta L}{l{\times S}_{L}}\right)}^{2}+{\left(\frac{\Delta C_{\mathrm{ab}}}{c\times S_{C}}\right)}^{2}+{\left(\frac{\Delta H_{\mathrm{ab}}}{S_{H}}\right)}^{2}}$$

$$T=0.56+\left|0.2\cos\left(h_{1}+168{}^{\circ}\right)\right|\;(\mathrm{if}164{}^{\circ}\leq h_{1}\leq 345{}^{\circ});$$

(18) $$T=0.36+\left|0.4\cos\left(h_{1}+35{}^{\circ}\right)\right|\;(\mathrm{otherwise})\;$$

(19) $$F=\sqrt{\frac{C_{1}^{4}}{C_{1}^{4}+\;1900}}$$

(20) $$S_{L}=0.511\;(\mathrm{if}L_{1}<16);S_{L}=\frac{0.040975\;L_{1}}{1+0.01765\;L_{1}}(\mathrm{if}L_{1}\geq 16)$$

(21) $$S_{C}=\frac{0.0638\;C_{1}}{1+0.0131\;C_{1}}+0.0638$$

(22) $$S_{H}=S_{C}\left(F\times T+1-F\right)$$

### 2.8 ColorI-DT

**Fig. 2:**
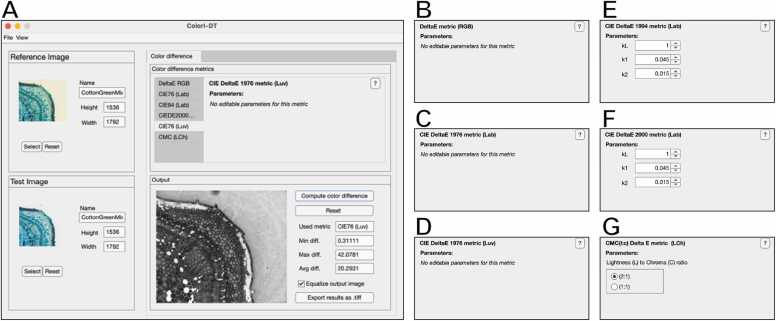
*ColorI-DT* main GUI (**A**) and specific windows for setting the parameters of the different metrics implemented: (**B**) Euclidean ΔE; (**C**) CIE76 (Lab); (**D**) CIE76 (Luv); (**E**) CIE94; (**F**) CIE00; (**G**) CMC.

*ColorI-DT* is an open-source tool developed in MATLAB R2022b (The MathWorks, Inc., Natick, MA, USA), available also as a standalone executable (*i.e*., not requiring MATLAB being installed) for *Windows 10*, *MacOS 13* (Ventura), *Ubuntu Linux 20.04*, or superior operating systems. *ColorI-DT* implements the 6 color difference metrics reported in Section 2.1 (Fig. 2**a**). Briefly, it allows to easily compute the pixel-wise difference between a pair of color images by: (*a*) converting the images to the target color space (*e*.*g*., CIELAB, CIELUV), (*b*) performing the pixel-wise calculation for the selected metric and parameters (Fig. 2**b-g**), (*c*) visualizing the results as a grayscale image of the same size as the input images, (*d*) saving the output as a 2D error matrix in “.txt” format, as well as a “.tiff” 8-bit image. The source code and compiled standalone versions are freely provided at: https://sourceforge.net/p/colori-dt/, together with user documentation, video tutorial and sample datasets.

### 2.9 Testing datasets

**Fig. 3:**
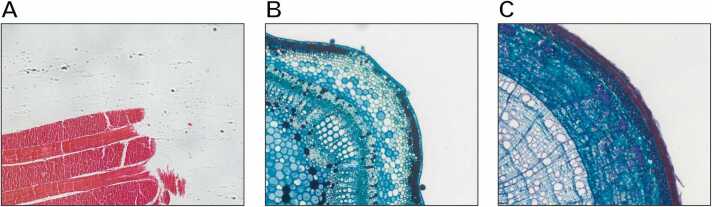
Original images used in the experiments: (**A**) a human tongue with predominant red colors (*Dataset A*); (**B**) a cotton stem with predominant green colors (*Dataset B*); (**C**) a wood stem with predominant blue colors (*Dataset C*).

Representative brightfield microscopy images with different predominant color palettes were used to test the tool. In particular, 3 different images (each one with a predominant color in one of the 3 different RGB channels) were acquired using 2 different microscopes: *(I)* an inverted brightfield Olympus (Tokyo, Japan) IX51 microscope endowed with a Nikon (Tokyo, Japan) Digital Sight DS-Vi1 color camera for transmitted light and a 10 × magnification objective has been used for acquiring a 1600 × 1200 pixel-size image of (*a*) an anatomical commercial histological slide (Salmoiraghi & Viganò, Legnago, Veneto, Italy) of a human tongue with predominant red colors (*Dataset A*, Fig. 3**a**); *(II)* an upright Zeiss (Oberkochen, Germany) Axioskop endowed with a Microvisioneer Basler (Esslingen and Ahrensburg, Germany) acA2040–55uc color camera for transmitted light and a 10 × magnification objective has been used for acquiring 1792 × 1536 pixel-size images of botanical commercial slides with sections of (*b*) a cotton stem with predominant green colors (*Dataset B*, Fig. 3**b**), and (*c*) a dicotyledonous wood stem with predominant blue colors (*Dataset C*, Fig. 3**c**).

**Fig. 4:**
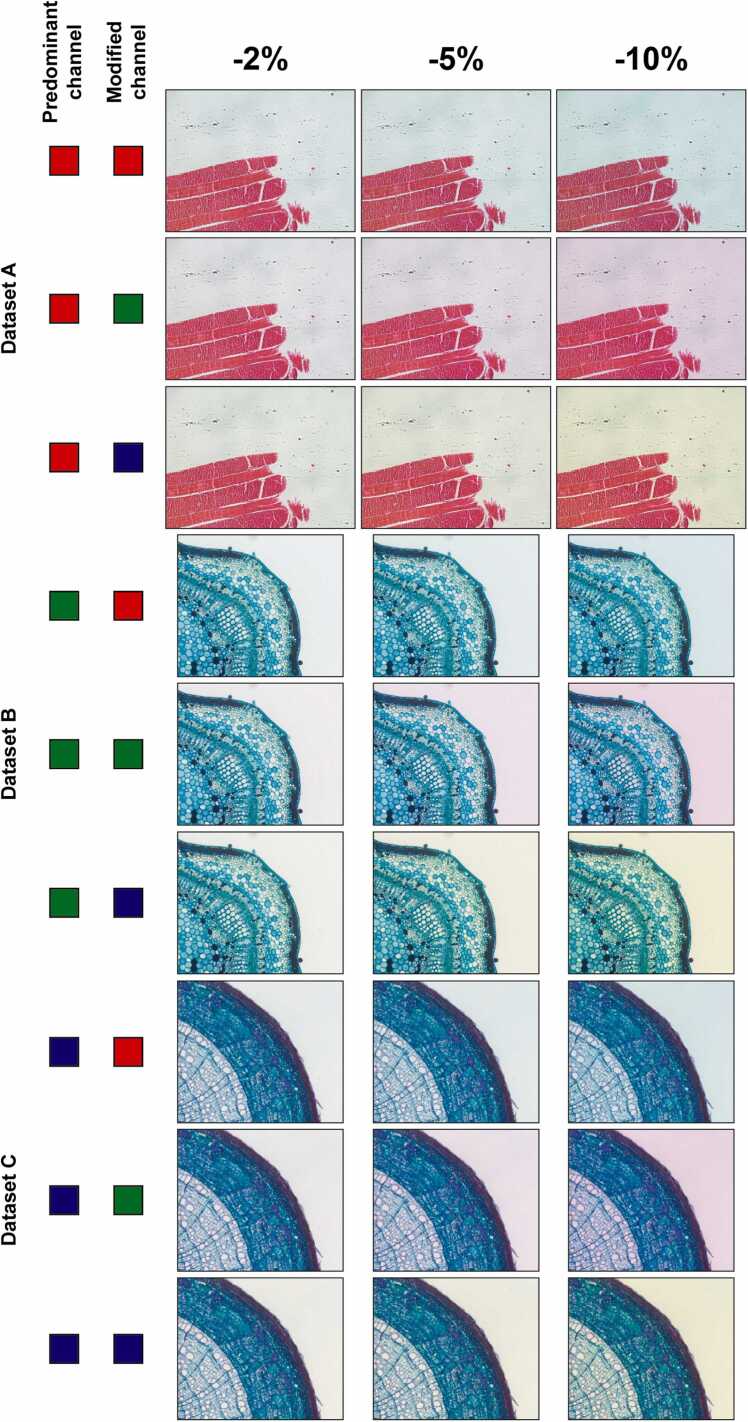
Synthetic images used in the experiments. Images of *Dataset A*, *B* and *C* are reported from top to down, respectively. The predominant RGB channel and the modified one are schematically shown on the left side of the figure.

To test the tool we investigated what happens when the color palette of the images is slightly altered, experiments were performed modifying the predominant channel of each image. In particular, each original image shown in Fig. 3 was used to generate 3 synthetic images in the red, green and blue channels separately, by subtracting 2 %, 5 % and 10 % intensity of each pixel. This resulted in a set of 9 altered images per original one, for a total of 27 synthetic images (Fig. 4).

All the images used in the experiments and shown in Fig. 3 and Fig. 4 are freely provided in full-size “.tif” format at: https://sourceforge.net/p/colori-dt/.

## 3 Results and discussion

### 3.1 Principal components analysis (PCA)

**Fig. 5:**
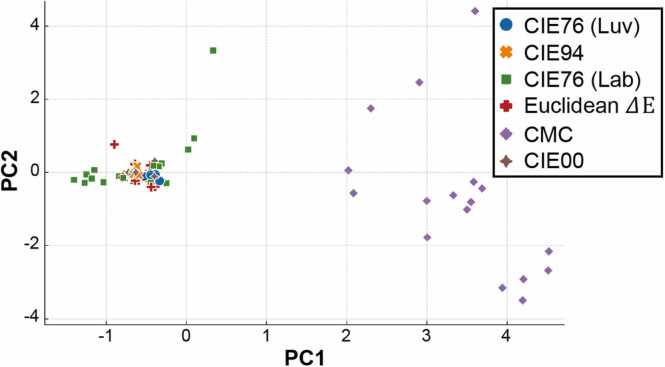
Detail of the PCA biplot. Legend: red crosses - Euclidean ΔE, green squares - CIE76 (Lab), blue dots - CIE76 (Luv), orange crosses - CIE94, brown crosses - CIE00, violet rhombuses - CMC, PC1 - principal component 1, PC2 - principal component 2.

Histogram statistics (*e.g*., mean, variance, skewness, kurtosis, and entropy) were computed for the 27 2D error matrices obtained for each metrics, for a total of 162 2D error matrices analyzed. Euclidean ΔE was used as reference for scalability and to assess linear dependencies among the other metrics. PCA of the 162 histograms was performed to examine, in a classical biplot, the variance within and between the clusters obtained for the 6 different metrics (Fig. 5). A well-defined cluster in the 2D plane suggests a homogeneous group, indicating that the observations are very similar to each other, meaning the characteristics represented by the principal components are very consistent. Conversely, an open cluster indicates great variability among the observations, suggesting a more heterogeneous group. Accordingly, a reliable metric should generate a well-defined cluster for the observations, as they were obtained corrupting a single primary RGB channel by a limited percentage.

The first PCA component (PC1) shows strong loadings on mean and variance, likely capturing aspects related to overall image brightness and contrast. The second PCA component (PC2) has strong loadings on skewness and kurtosis, capturing the shape of the gray-level distribution. PCA analysis reveals well-defined clusters for all metrics, except for CMC and CIE76 (Lab), which exhibit significant dispersion.

### 3.2 Statistics and channel variations correlations

**Fig. 6:**
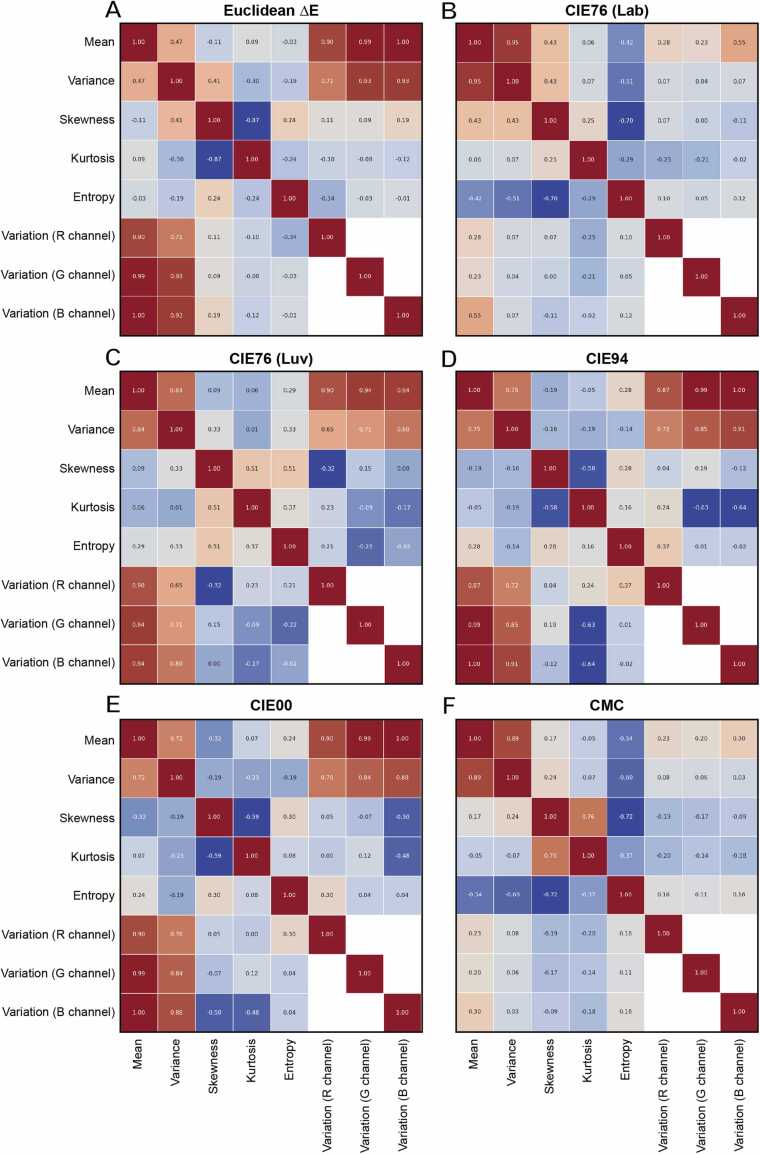
Correlation heatmaps for the different metrics: (**A**) Euclidean ΔE; (**B**) CIE76 (Lab); (**C**) CIE76 (Luv); (**D**) CIE94; (**E**) CIE00; (**F**) CMC.

Correlation heatmaps were then computed by correlating histogram statistics of the 162 2D error matrices and variations of the primary channels (Fig. 6).

Among the different variables, mean and variance showed in general the strongest positive correlations for all the metrics. Therefore, the analysis primarily focused on these variables. Once again, output 8-bit grayscale images obtained for CMC and CIE76 (Lab) behave as outliers, displaying the weakest correlations (represented with blue/cyan/white colors) between variations of the primary channels and the average values across the heatmap board.

### 3.3 Linear regression analysis

**Fig. 7:**
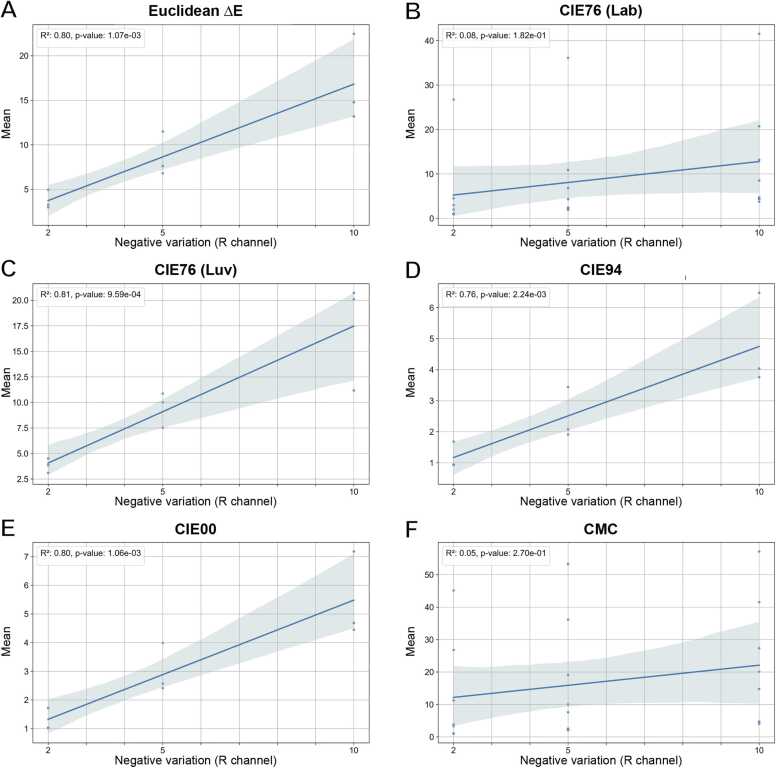
Linear regression analysis considering mean and variation of the R channel: (**A**) Euclidean ΔE; (**B**) CIE76 (Lab); (**C**) CIE76 (Luv); (**D**) CIE94; (**E**) CIE00; (**F**) CMC.

The metric with the most linear relationship with channel variations should be the favorite when dealing with limited color alterations. Accordingly, a linear regression analysis was performed by considering mean (Fig. 7, Supplementary Figure 1, Supplementary Figure 2) and variance (Supplementary Figure 3, Supplementary Figure 4, Supplementary Figure 5) versus the different channel variations. The values related to the R channel are shown in Fig. 7 and Supplementary Figure 3, the ones related to the G channel in Supplementary Figure 1 and Supplementary Figure 4, and the ones of the B channel in Supplementary Figure 2 and Supplementary Figure 5. Pearson correlation coefficient (i.e. R^*2*^) and Wald *p*-value are directly reported in the different figures in the upper left corner of each plot. In addition, Table 1 reports for the different metrics (column 1) the: (column 2) average R^*2*^ of means; (column 3) average R^*2*^ of variances; (column 4) average slope of the linear fits of the means (with margins of error); (column 5) average intercept of the linear fits of the means (with margins of error).

**Table 1:** Linear regression analysis statistics.

**METRIC**	**AVERAGE R**^**2**^**OF MEANS**	**AVERAGE R**^**2**^**OF VARIANCES**	**AVERAGE SLOPE OF MEANS**	**AVERAGE INTERCEPT** **OF MEANS**
**Euclidean**Δ**E**	0.93	0.74	$1.79_{\left[-1.68\right]}^{\left[+1.90\right]}$	$0.45_{\left[-0.38\right]}^{\left[+0.52\right]}$
**CIE76 (Lab)**	0.15	0.01	$1.42_{\left[-0.75\right]}^{\left[+2.09\right]}$	$4.12_{\left[-3.85\right]}^{\left[+4.40\right]}$
**CIE76 (Luv)**	0.86	0.52	$1.84_{\left[-1.75\right]}^{\left[+1.92\right]}$	$1.00_{\left[-0.94\right]}^{\left[+1.05\right]}$
**CIE94**	0.92	0.69	$0.80_{\left[-0.75\right]}^{\left[+0.85\right]}$	$0.17_{\left[-0.14\right]}^{\left[+0.20\right]}$
**CIE00**	0.93	0.69	$0.77_{\left[-0.74\right]}^{\left[+0.81\right]}$	$0.43_{\left[-0.41\right]}^{\left[+0.45\right]}$
**CMC**	0.06	0. 01	$1.88_{\left[-1.15\right]}^{\left[+2.61\right]}$	$15.72_{\left[-15.43\right]}^{\left[+16.02\right]}$

Euclidean ΔE presented strong correlations and a linear behavior. This result was expected, as this metric does not involve any change in color coordinates, and the differences are defined within the same color space as the input images. In contrast, all other metrics require a transformation from RGB to CIELAB or CIELUV, and the non-linearities in these transformations or in the metrics themselves can impact scalability and linearity.

The first notable result is the divergent behavior of the CIE76 (Lab) and CMC metrics. For both metrics, the mean and variance showed weak correlations with the variations in the primary R, G, and B channels. CIE76 (Lab), essentially a Euclidean distance, might not be sensitive enough to small color variations due to the coordinate definition in the Lab color space. Similarly, CMC, also defined in the CIELAB color space, shows erratic behavior in evaluating color distance in case of bright white image background.

CIE94, CIE00, and CIE76 (Luv) showed strong correlations and linearities with the alterations in primary RGB channels. Compared with Euclidean ΔE, CIE94 and CIE00 displayed almost identical average R² values for the mean, around R² = 0.92, with CIE76 (Luv) slightly lower at R² = 0.86. Similarly, the R² values for the variance showed alignment between the results of CIE94 and CIE00, with again CIE76 (Luv) slightly lower. Similarly, the correlation values for the variance still show moderate correlations between the variance and the amount of variations in the different channels, although they are lower than those for the mean.

Finally, it is worth considering that in general the slope of the linear fits should intercept the origin of the Cartesian plane to show a correct linear relationship between mean/variance and channel variation, which corresponds to no change in channel values. The intercept values for all metrics are consistent with the expected zero value within their confidence intervals. Compared to Euclidean ΔE (used as the linearity benchmark), CIE76 (Luv) has the most similar average slope of means and CIE00 has the closest average intercept of means.

In conclusion, the linear regression analysis showed that in the test performed, among the analyzed metrics (excluding the non-perceptually accurate Euclidean ΔE), CIE00 and CIE94 achieved very similar results, with CIE76 (Luv) slightly behind but still demonstrating strong correlation and linearity with variations in primary RGB colors.

## 4 Conclusions and perspectives

In histopathology, color images are crucial for visualizing samples stained with specific stains. Colors helps pathologists in identifying cancer cells, inflammation, and other pathological conditions. However, since different operators may use various imaging equipment or illumination settings, color can vary significantly.

In this work, besides listing the metrics currently published for comparing color images, we presented *ColorI-DT*, an open-source tool for measuring quantitative differences between color images using one of the 6 following metrics: (*1*) Euclidean ΔE; (*2*) CIE76 (Luv); (*3*) CIE76 (Lab); (*4*) CIE94; (*5*) CIE00; (*6*) CMC. Thanks to *ColorI-DT* users can now: (*a*) visualize pairs of images; (*b*) select a color difference metric among the ones currently proposed in the literature; (*c*) adjust parameters (e.g., weighting functions in CIE94 or CIE00); (*d*) visualize the results in real-time; (*e*) export the 2D error matrix in formats such as “.txt” or grayscale “.tiff” images.

Despite metric revalidation was behind the aim of this work, brightfield microscopy images with a predominant color were used to test the tool. Specifically, while evaluating the various implemented features, we indirectly identified which metrics exhibited the most predictable and linear behavior in response to controlled primary color alterations applied to a reference image. CIE00 met CIE expectations as the recommended metric in the CIELAB color space and CIE94 produced similar results. CIE76 metrics are characterized by a simpler mathematical definition than the other metrics and share the same distance formula. However, it is interesting to note how CIE76 (Luv) outperformed CIE76 (Lab). As reported in previous literature [27] [28], this again suggests that the CIELAB color space, despite remains more used compared to CIELUV, is not truly uniform. In summary, the experiments performed showed that CIE76 (Luv), CIE94, and CIE00 are all strongly correlated to small, controlled alterations in the primary color channel, with a preference for the most recent CIE00. However, thanks to *ColorI-DT*, researchers and clinicians now have at their disposal a tool for easily comparing color images, regardless of the metric they choose to use.

Concluding, this work provides the scientific community with: (*1*) the first open-source library containing the currently published color evaluation metrics; (*2*) an open-data testbed for easily testing future metrics on slight primary color alterations; (*3*) the first freely available tool for easily quantitatively comparing color images. Although no new quantitative metric has been proposed, *ColorI-DT* makes state-of-the-art metrics accessible to the community. However, several future directions may enhance *ColorI-DT*’s applicability and performance. For instance, the analysis can be extended across a wider range of imaging modalities, including fluorescence and electron microscopy, as well as natural scenes, industrial images, and digital artwork, to assess the robustness and versatility of the metrics in specific cases.

## Ethics statement

This study does not involve human subjects, human samples, or any procedures requiring ethical approval. Therefore, no ethical approval was required for this research.

## Funding

N.N. acknowledges support from the Italian Ministry of Health and the contribution of “Ricerca Corrente” within the research line “Appropriateness, outcomes, drug value and organisational models for the continuity of diagnostic therapeutic pathways in oncology”. F.P. and G.C. acknowledge the support received from the MAECI Science and Technology Cooperation Italy–South Korea Grant Years 2023–2025 by the Italian Ministry of Foreign Affairs and International Cooperation (CUP project: J53C23000300003); J.-C.P. and M.L. from the 10.13039/501100001321National Research Foundation (Funding No.: 2022K1A3A1A25081295).

## CRediT authorship contribution statement

**Michele Tritto:** Writing – original draft, Visualization, Software, Methodology, Investigation, Formal analysis, Data curation, Conceptualization. **Jae-Chul Pyun:** Writing – review & editing, Validation, Supervision, Resources, Funding acquisition, Data curation. **Misu Lee:** Writing – review & editing, Validation, Investigation, Conceptualization. **Bongseop Kwak:** Writing – review & editing, Visualization, Formal analysis, Data curation, Conceptualization. **Bosung Ku:** Writing – original draft, Visualization, Validation, Resources, Formal analysis, Conceptualization. **Nicola Normanno:** Writing – review & editing, Supervision, Resources, Funding acquisition. **Gastone Castellani:** Writing – original draft, Supervision, Software, Resources, Investigation, Funding acquisition. **Filippo Piccinini:** Writing – original draft, Visualization, Software, Resources, Project administration, Methodology, Investigation, Funding acquisition, Formal analysis, Data curation, Conceptualization.

## Declaration of Competing Interest

M.T. was employed by the Next2U Srl, Pescara, Italy. B.Ku was employed by the Central R&D Center, Medical and Bio Decision (MBD) Co., Ltd, Suwon, Republic of Korea. The remaining authors declare that the research was conducted in the absence of any commercial or financial relationships that could be construed as a potential conflict of interest.

## GLOSSARY

CIE00: A color difference metric using the Euclidean distance in the CIELAB color space, introduced by the International Commission on Illumination (CIE) in 2000.
CIE76 (Lab): A color difference metric using the Euclidean distance in the CIELAB color space, introduced by the International Commission on Illumination (CIE) in 1976.
CIE76 (Luv): A color difference metric using the Euclidean distance in the CIELUV color space, introduced by the International Commission on Illumination (CIE) in 1976.
CIE94: A color difference metric using the Euclidean distance in the CIELAB color space, introduced by the International Commission on Illumination (CIE) in 1994.
CIELAB: A color space representing colors more uniformly in terms of human vision.
CIELUV: A color space useful for additive color mixtures.
CMC (LCh): A color difference metric developed by the Colour Measurement Committee of the Society of Dyers and Colourists. It uses a cylindrical representation of the CIELAB space and applies weighting functions based on lightness, chroma, and hue.
ColorI-DT: An open-source tool developed to measure quantitative differences between color images.
Euclidean Delta E: A color difference metric using the Euclidean distance in the RGB color space, simple but not perceptually accurate.
LCh: A cylindrical representation of the CIELAB space, where L stands for lightness, C for chroma, and h for hue.
PCA: A statistical technique used to reduce the dimensionality of data while preserving as much variability as possible by transforming the original variables into a new set of uncorrelated variables called principal components.
Pixel-wise Color Difference Matrix: A 2D matrix that displays the color difference values for corresponding pixels in two images.
Quantitative Color Comparison: The process of numerically comparing color differences between images.
RGB Color Space: A color model using the primary colors red, green, and blue, commonly used in digital imaging.

## References

[1] Chandler D.M. (2013). Seven challenges in image quality assessment: past, present, and future research. Int Sch Res Not.

[2] Roy S., Kumar Jain A., Lal S., Kini J. (2018). A study about color normalization methods for histopathology images. Micron.

[3] Nsoesie E.O. (2018). Evaluating artificial intelligence applications in clinical settings. JAMA Netw Open.

[4] Piccinini F., Bevilacqua A., Lucarelli E. (2013). Automated image mosaics by non-automated light microscopes: the MicroMos software tool. J Microsc.

[5] Chai T., Draxler R.R. (2014). Root mean square error (RMSE) or mean absolute error (MAE). Geosci Model Dev Discuss.

[6] Box G. (1988). Signal-to-noise ratios, performance criteria, and transformations. Technometrics.

[7] Wang Z., Bovik A.C. (2002). A universal image quality index. IEEE Signal Process Lett.

[8] Piccinini F., Balassa T., Carbonaro A., Diosdi A., Toth T., Moshkov N., Tasnadi E.A., Horvath P. (2020). Software tools for 3D nuclei segmentation and quantitative analysis in multicellular aggregates. Comput Struct Biotechnol J.

[9] Bevilacqua A., Gherardi A., Piccinini F. Quantitative quality assessment of microscopic image mosaicing, International Journal of Electronics and Communication Engineering 2010; 4(11):1608-1611.

[10] Tremeau A., Tominaga S., Plataniotis K. (2008). Color in image and video processing: most recent trends and future research directions. EURASIP J Image Video Process.

[11] Piccinini F., Tazzari M., Tumedei M.M., Stellato M., Remondini D., Giampieri E., Martinelli G., Castellani G., Carbonaro A. (2024). Data Science for Health Image Alignment: A User-Friendly Open-Source ImageJ/Fiji Plugin for Aligning Multimodality/Immunohistochemistry/Immunofluorescence 2D Microscopy Images. Sensors.

[12] Ansari M., Singh D.K. (2022). Significance of color spaces and their selection for image processing: a survey. Recent Advances Computer Science Communications (Formerly Recent Patents Computer Science).

[13] Süsstrunk S., Buckley R., Swen S. (1999). Standard RGB color spaces, In Color and imaging conference. Soc Imaging Sci Technol.

[14] Wang L., Li C., Xu Y., Melgosa M., Xiao K., Gao K. (2023). Improvements to the CIELUV color space. Color Res Appl.

[15] Ohta N. (1977). Correspondence between CIELAB and CIELUV color differences. Color Research Application Endorsed Inter-Society Color Council Colour Group (Great Britain) Canadian Society Color Color Science Association Japan Dutch Society Study Color Swedish Colour Centre Foundation Colour Society Australia Centre Français de la Couleur.

[16] Sarifuddin M., Missaoui R. (2005). A new perceptually uniform color space with associated color similarity measure for content-based image and video retrieval. Proc ACM SIGIR 2005 Workshop Multimed Inf Retr (MMIR).

[17] Lissner I., Urban P. (2011). Toward a unified color space for perception-based image processing. IEEE Trans Image Process.

[18] Munkres J. Topology: 2nd edition. Pearson Education Limited, Edinburgh Gate. (2000). ISBN: 978-1-292-02362-5.

[19] Robertson A.R. (1990). Historical development of CIE recommended color difference equations. Color Research Application Endorsed Inter-Society Color Council Colour Group (Great Britain) Canadian Society Color Color Science Association Japan Dutch Society Study Color Swedish Colour Centre Foundation Colour Society Australia Centre Français de la Couleur.

[20] McDonald R., Smith K.J. (1995). CIE94-a new colour-difference formula. J Soc Dyers Colour.

[21] Klein G.A., Meyrath T. Industrial color physics. New York: Springer. 2010; 154. ISBN: 9781461426028.

[22] Alman D.H., Berns R.S., Chong T.F., Hita E., Kehlibarov T., Komatsubara H., Maier T.O., MacDonald R., Reilly C.D., Robertson A.R., Sève R., Shah H.S., Smith K.J., Witt K.C.I.E., Technical report: Industrial colour-difference evaluation 1995; 116. ISBN: 9783900734602.

[23] Melgosa M., Quesada J.J., Hita E. (1994). Uniformity of some recent color metrics tested with an accurate color-difference tolerance dataset. Appl Opt.

[24] Luo M.R., Cui G., Rigg B. (2001). The development of the CIE 2000 colour-difference formula: CIEDE2000. Color Research Application Endorsed Inter-Society Color Council Colour Group (Great Britain) Canadian Society Color Color Science Association Japan Dutch Society Study Color Swedish Colour Centre Foundation Colour Society Australia Centre Français de la Couleur.

[25] Mokrzycki W.S., Tatol M. (2011). Colour difference Delta E - A survey. Mach Graph Vis.

[26] Heggie D., Wardman R.H., Luo M.R. (1996). A comparison of the colour differences computed using the CIE94, CMC (l: c) and BFD (l: c) formulae. J Soc Dyers Colour.

[27] Moroney N. (2003). A hypothesis regarding the poor blue constancy of CIELAB. Color Research Application Endorsed Inter-Society Color Council Colour Group (Great Britain) Canadian Society Color Color Science Association Japan Dutch Society Study Color Swedish Colour Centre Foundation Colour Society Australia Centre Français de la Couleur.

[28] Sharma G., Rodríguez-Pardo C. (2012). The dark side of CIELAB. Proc Color Imaging XVII Disp Process Hardcopy Appl SPIE Int Soc Opt Eng.

