# Supplementary material for: ColorI-DT: An open-source tool for the quantitative evaluation of differences in microscopy color images
Source: Comput Struct Biotechnol J. 2025 Jun 9;27:2526–36. doi: 10.1016/j.csbj.2025.06.019 (PMC12197881; doi:10.1016/j.csbj.2025.06.019)

**Supplementary Note 1**

*MATLAB* implementation for the different metrics.

function [diff, m, M, ave] = deRGB(rImg,tImg)

%This function calculates dE in rgb space.

if size(rImg,3) == 4

rImg=rImg(:,:,1:3);

end

if size(tImg,3) ==4

tImg=tImg(:,:,1:3);

end

diff = sqrt(sum((tImg-rImg).^2,3));

m = min(diff, [], "all");

M = max(diff, [], "all");

ave = mean(diff, "all");

return

end

**Code 1**: *MATLAB* source code of the metric Euclidean $\Delta E$ (RGB).

function [diff, m, M ,ave] = luv76(rImg, tImg)

% This fuction is an original implementation of the CIEDE76 color difference

% in LUV space.

% It calculates the difference between the reference image "rImg" and the target

% image "tImg" and stores it in the "diff" matrix of which calculates the

% minimum, the maximum and the average value of intensity.

if size(rImg,3) == 4

rImg=rImg(:,:,1:3);

end

if size(tImg,3) ==4

tImg=tImg(:,:,1:3);

end

rImg = rgb2luv(rImg);

tImg = rgb2luv(tImg);

diff = sqrt(sum((tImg-rImg).^2, 3));

m = min(diff, [], "all");

M = max(diff, [], "all");

ave = mean(diff, "all");

return

end

**Code 2**: *MATLAB* source code of the metric CIE76 (Luv).

function [diff, m, M, ave] = lab76(rImg, tImg)

% This function implements CIEDE76 (L*a*b*).

% It calculates the difference between the reference image rImg and the target

% image tImg and stores it in the "diff" matrix of which calculates the

% minimum, the maximum and the average value of intensity.

if size(rImg,3) == 4

rImg=rImg(:,:,1:3);

end

if size(tImg,3) ==4

tImg=tImg(:,:,1:3);

end

rImg = rgb2lab(rImg);

tImg = rgb2lab(tImg);

diff = sqrt(sum((tImg-rImg).^2, 3));

m = min(diff, [], "all");

M = max(diff, [], "all");

ave = mean(diff, "all");

return

end

**Code 3**: *MATLAB* source code of the metric CIE76 (Lab).

function [diff, m, M, ave] = lab94(rImg,tImg, kL, k1, k2)

% This function invokes the CIE94 in LAB color space as implemented by

% "imcolordiff" function in MATLAB.

% It calculates the difference between the reference image rImg and the target

% image tImg and stores it in the "diff" matrix, taking as parameters the

% values "kL", "k1", "k2" (for more details reference the help page of

% "imcolordiff").

if size(rImg,3) == 4

rImg=rImg(:,:,1:3);

end

if size(tImg,3) ==4

tImg=tImg(:,:,1:3);

end

rImg = rgb2lab(rImg);

tImg = rgb2lab(tImg);

diff = imcolordiff(rImg, tImg,"Standard","CIE94","isInputLab",true,"kL",kL,"K1" ,k1 ,"K2" ,k2);

m = min(diff, [], "all");

M = max(diff, [], "all");

ave = mean(diff, "all");

return

end

**Code 4**: *MATLAB* source code of the metric CIE94 (Lab).

function [diff, m, M, ave] = lab00(rImg,tImg, kL, k1, k2)

% This unction invokes the CIEDE2000 in LAB color space as implemented by

% "imcolordiff" function in MATLAB.

% It calculates the difference between the reference image rImg and the target

% image tImg and stores it in the "diff" matrix, taking as parameters the

% values "kL", "k1", "k2" (for more details reference the help page of

% "imcolordiff").

% "diff" it calculates the minimum, the maximum and the average value of intensity.

if size(rImg,3) == 4

rImg=rImg(:,:,1:3);

end

if size(tImg,3) ==4

tImg=tImg(:,:,1:3);

end

rImg = rgb2lab(rImg);

tImg = rgb2lab(tImg);

diff = imcolordiff(rImg, tImg,"Standard","CIEDE2000","isInputLab",true,"kL", kL ,"K1",k1 ,"K2",k2);

m = min(diff, [], "all");

M = max(diff, [], "all");

ave = mean(diff, "all");

return

end

**Code 5**: *MATLAB* source code of the metric CIE00 (Lab).

function [diff, m, M ,ave] = lchcmc(rImg, tImg, ratio)

%Implementation of the CMC (l:c) color difference metric in LCh lab color space.

if size(rImg,3) == 4

rImg=rImg(:,:,1:3);

end

if size(tImg,3) ==4

tImg=tImg(:,:,1:3);

end

rImg = rgb2lch(rImg);

tImg = rgb2lch(tImg);

% assign l, c values based on passed ratio

if isequal(ratio,2)

l=2;

c=1;

else

l=1;

c=1;

end

L1 = rImg(:,:,1);

L2 = tImg(:,:,1);

C1 = rImg(:,:,2);

C2 = tImg(:,:,2);

h1 = rImg(:,:,3);

h2 = tImg(:,:,3);

dL=L2-L1;

dC=C2-C1;

dh=h2-h1;

%F, T

F=sqrt(((C1).^4)./((C1.^4)+1900));

if 164<=h1 && h1<=145

T=0.56+abs(0.2*cosd(h1+168));

else

T=0.36+abs(0.4*cosd(h1+35));

end

%S_L

if L1<16

S_L =0.511;

else

S_L = (0.040975*L1)./(1+0.01765*L1);

end

%S_C

S_C = ((0.0638*C1)./(1+0.0131*C1)) +0.638;

%S_H

S_H = S_C.*(F.*T +1 - F);

%definition of the deCMC color difference formula

diff = sqrt(((dL./(l*S_L)).^2+(dC./(c*S_C)).^2+(dh./S_H).^2));

m = min(diff, [], "all");

M = max(diff, [], "all");

ave = mean(diff, "all");

return

end

**Code 6**: *MATLAB* source code of the metric CMC (LCh).

**Supplementary Figure 1**

Linear regression analysis considering mean and variation of the G channel: (**A**) Euclidean $\Delta E$; (**B**) CIE76 (Lab); (**C**) CIE76 (Luv); (**D**) CIE94; (**E**) CIE00; (**F**) CMC.

**
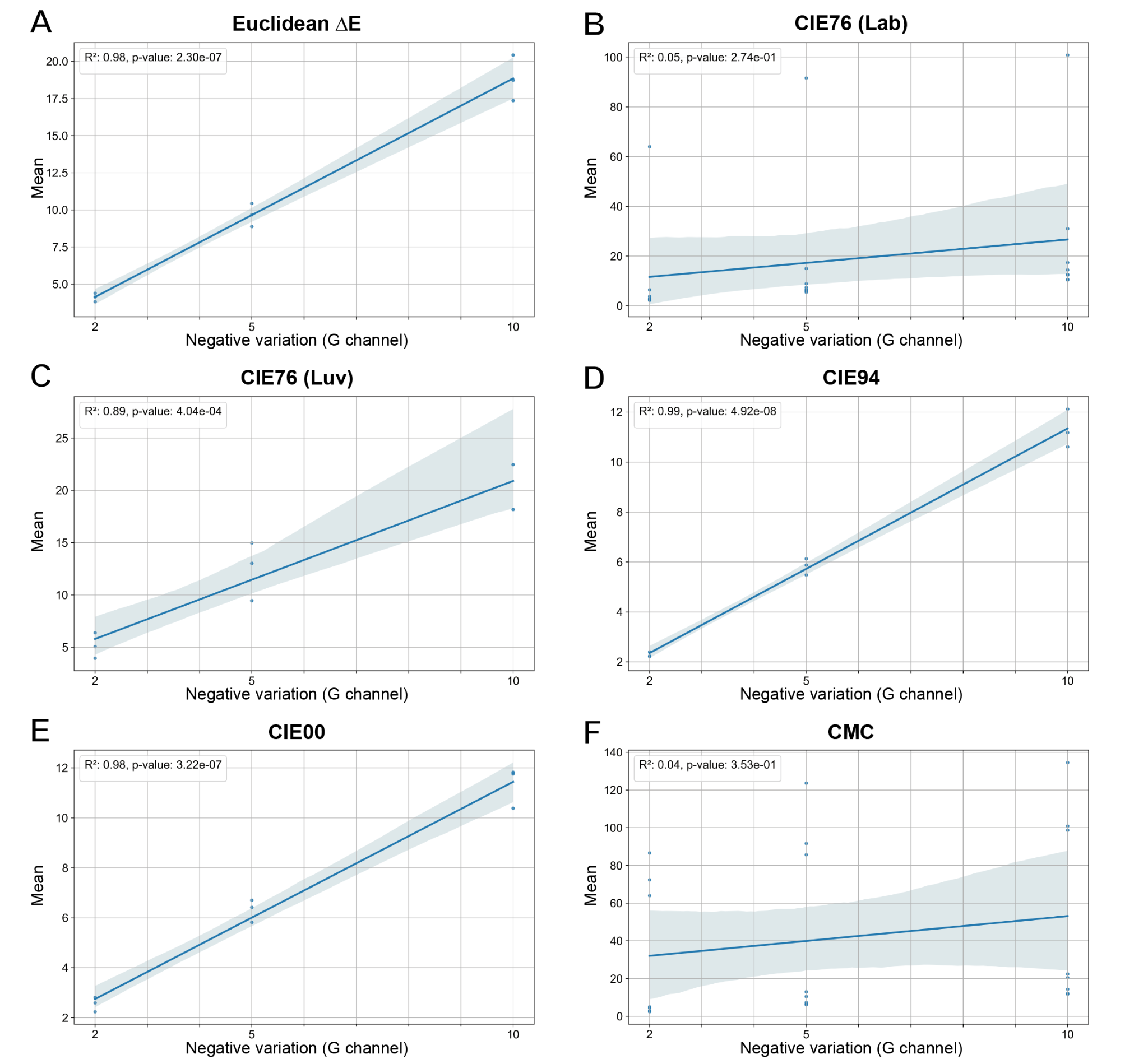
**

**Supplementary Figure 2**

Linear regression analysis considering mean and variation of the B channel: (**A**) Euclidean $\Delta E$; (**B**) CIE76 (Lab); (**C**) CIE76 (Luv); (**D**) CIE94; (**E**) CIE00; (**F**) CMC.


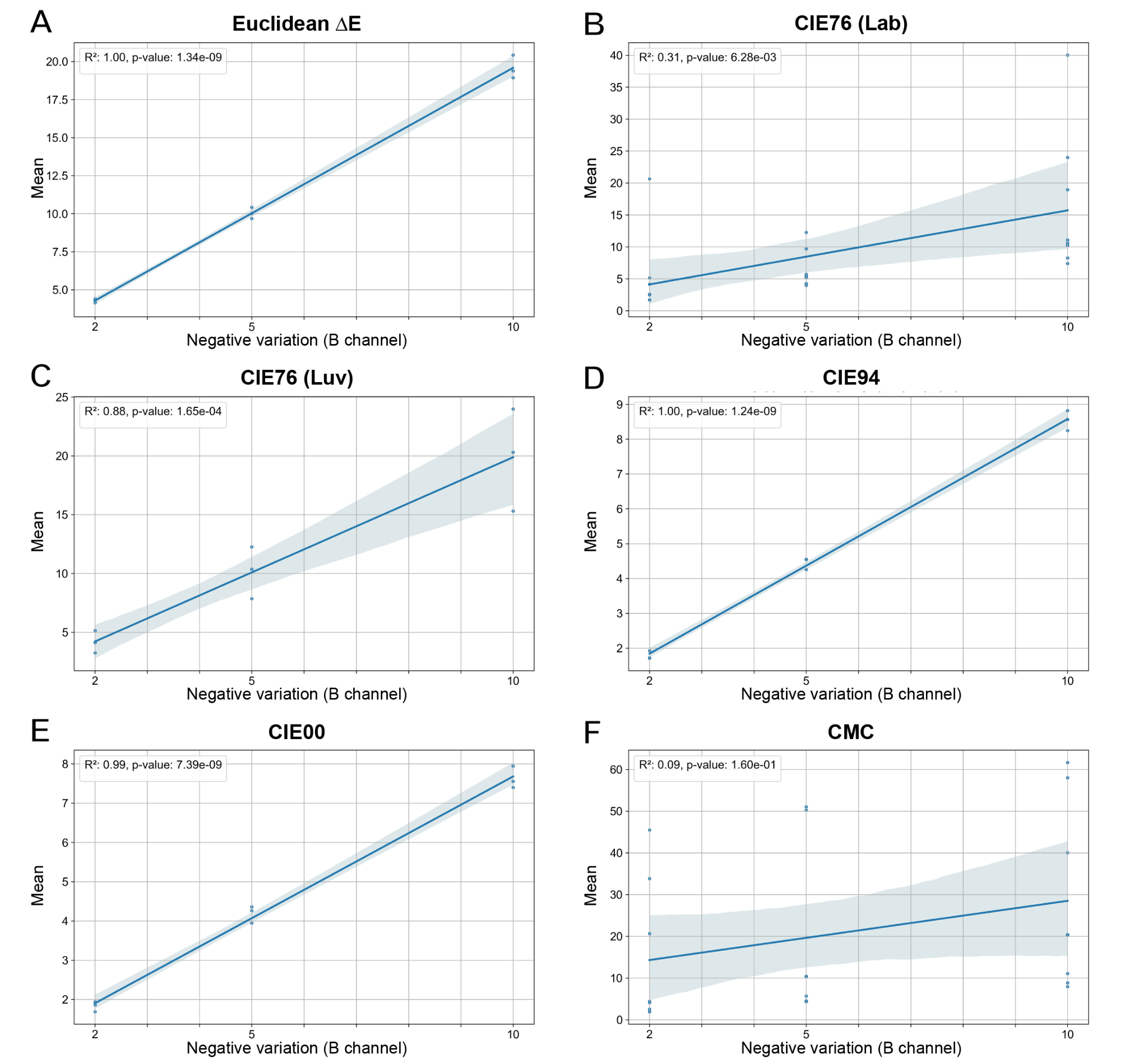


**Supplementary Figure 3**

Linear regression analysis considering variance and variation of the R channel: (**A**) Euclidean $\Delta E$; (**B**) CIE76 (Lab); (**C**) CIE76 (Luv); (**D**) CIE94; (**E**) CIE00; (**F**) CMC.


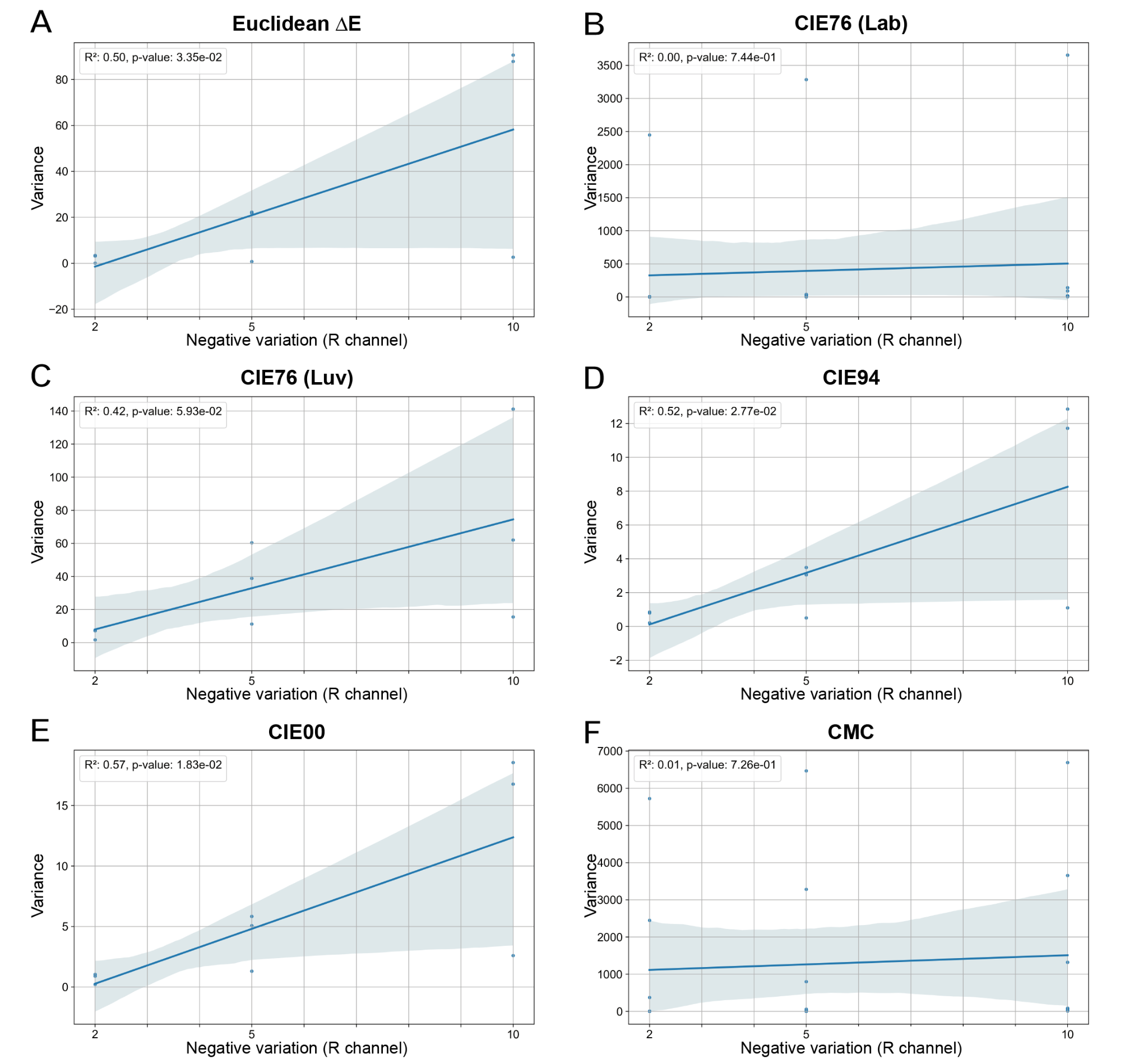


**Supplementary Figure 4**

Linear regression analysis considering variance and variation of the G channel: (**A**) Euclidean $\Delta E$; (**B**) CIE76 (Lab); (**C**) CIE76 (Luv); (**D**) CIE94; (**E**) CIE00; (**F**) CMC.


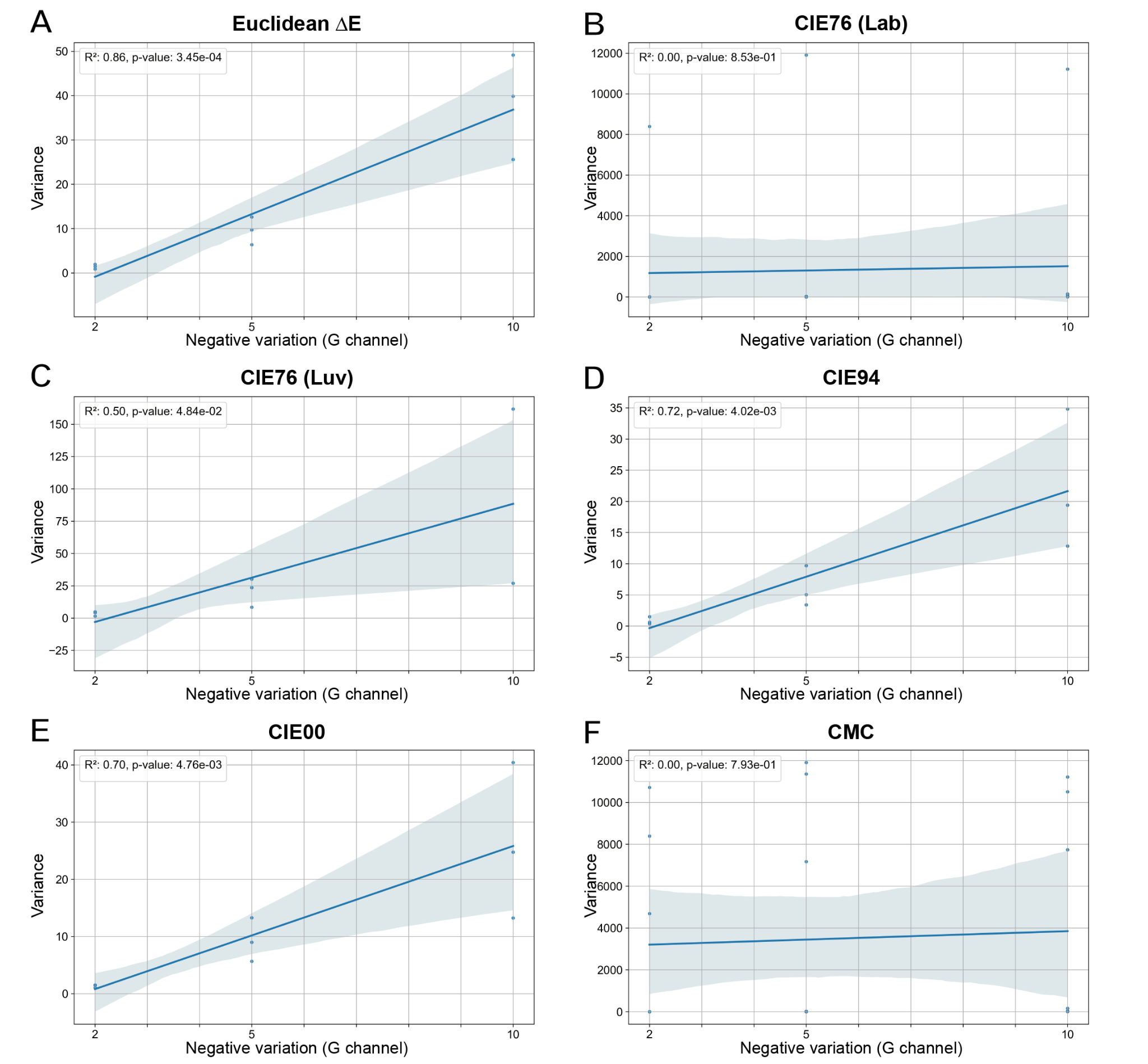


**Supplementary Figure 5**

Linear regression analysis considering variance and variation of the B channel: (**A**) Euclidean $\Delta E$; (**B**) CIE76 (Lab); (**C**) CIE76 (Luv); (**D**) CIE94; (**E**) CIE00; (**F**) CMC.


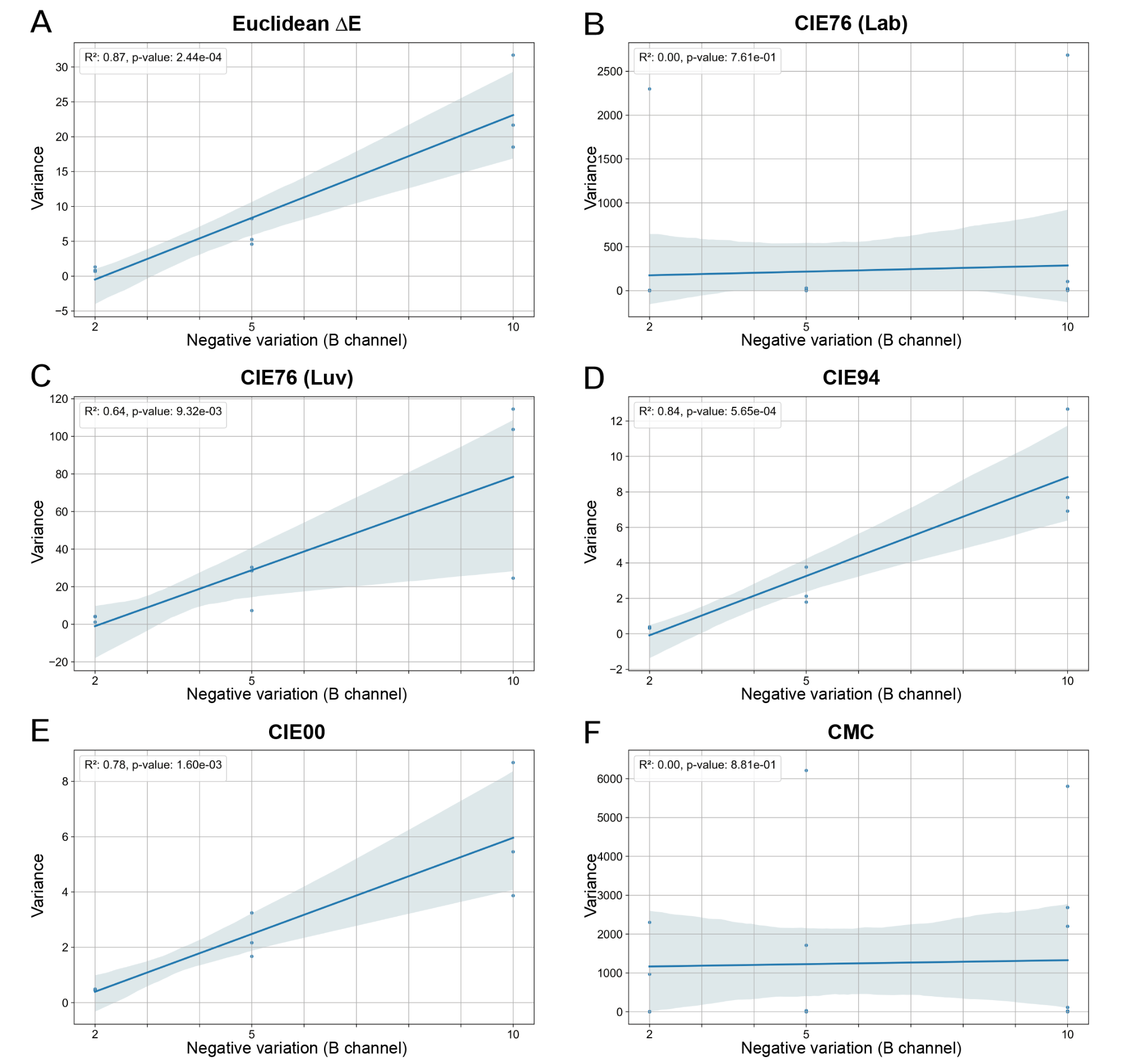

Supplement: Supplementary Figure 1 — Supplementary material [file mmc1.docx]
